# Fundus Blood Flow in Patients with Sellar Lesions with Optic Nerve Bending and Chiasmal Compression

**DOI:** 10.3390/jcm14165790

**Published:** 2025-08-15

**Authors:** Yoichiro Shinohara, Rei Yamaguchi, Masahiko Tosaka, Soichi Oya, Hideo Akiyama

**Affiliations:** 1 Department of Ophthalmology, Gunma University Graduate School of Medicine, Gunma 371-8511, Japan; 2 Department of Neurosurgery, Gunma University Graduate School of Medicine, Gunma 371-8511, Japan; rei.yamaguchi@gunma-u.ac.jp (R.Y.); sooya-gnm@gunma-u.ac.jp (S.O.)

**Keywords:** optic nerve bending, sellar lesions, laser speckle flowgraphy, pituitary neuroendocrine tumor, ganglion cell, optical coherence tomography

## Abstract

**Background/objectives**: Optic nerve bending and chiasmal compression impair vision in patients with sellar lesions; however, their effect on optic nerve head (ONH) blood flow remains unclear. This study used laser speckle flowgraphy to examine the relationship between clinical features and ONH blood flow in patients with optic nerve bending and chiasmal compression. **Methods**: This retrospective study included 32 eyes (16 eyes with and 16 without optic nerve bending on the contralateral side) from 16 patients with sellar lesions. The best-corrected visual acuity (BCVA), simple visual field impairment score (SVFIS), optic nerve head mean blur rate (ONH-MBR), and six-segmented macular ganglion cell layer + inner plexiform layer (GCL + IPL) thickness were examined. **Results**: Preoperative BCVA and SVFIS in eyes with optic nerve bending were significantly worse than those in eyes without bending, and significantly correlated with the optic nerve-canal bending angle (ONCBA). After tumor resection, BCVA and SVFIS significantly improved in both groups. Preoperative ONH-MBR was significantly lower in bending eyes but increased significantly post-treatment in both groups. Preoperative ONH-MBR correlated with ONCBA, while postoperative ONH-MBR correlated with nasal GCL + IPL thickness. **Conclusions**: Optic nerve bending and chiasmal compression showed reduced blood flow to the ONH. These changes in blood flow may be associated with GCL + IPL thickness and optic nerve bending angle.

## 1. Introduction

Pituitary neuroendocrine tumor (PitNET) and other tumors in the suprasellar and sellar regions can cause visual field defects, including bilateral hemianopsia, due to the compression of the optic chiasm by the tumor [1]. Many sellar tumors originate from the pituitary gland and pituitary stalk, progressing upward from the midline skull base [2], compressing the crossing fibers of the optic chiasm. The prevalence of PitNET ranges from 7.0 to 41.3 per 100,000 individuals, with an incidence of 0.65 to 2.34 per 100,000, and approximately 70% of patients with PitNET experience visual symptoms [3,4]. As the tumor grows, visual field defects caused by the compression of the optic chiasm may progress, resulting in visual impairment [5,6]. Other types of tumors in sellar regions, such as craniopharyngioma, Rathke’s cleft cyst, and meningioma, have different growth patterns and therefore show some variation in the pattern of compression on the optic chiasm, but they often cause hemianopia [7,8,9]. However, in clinical practice, patients with sellar tumors may lack bilateral hemianopsia despite having severe visual impairment. We previously investigated patients with sellar lesions who had visual impairment using magnetic resonance imaging (MRI), and reported that optic nerve bending at the canal entrance may contribute to visual loss [10]. We also reported that eyes with decreased ganglion cell layer + inner plexiform layer (GCL + IPL) thickness due to optic nerve bending caused by sellar tumors showed little improvement in visual acuity, even after tumor resection [11]. Recently, another group reported that optic nerve bending from sellar tumors is linked to visual impairment [12]. Therefore, a detailed understanding of the pathophysiological characteristics of optic nerve bending is essential for elucidating the mechanisms and characteristics of visual dysfunction in sellar tumors and for determining the optimal treatment timing.

Optic nerve compression and bending from sellar lesions can lead to demyelination and impaired blood flow in the optic nerve. Some reports have examined retinal vascular density in PitNET compressing the optic chiasm using optical coherence tomography angiography (OCTA) and predicted postoperative visual prognosis [13,14,15]. Although compression of the optic chiasm by brain tumors can induce loss of ganglion cells and cause changes in the microvascular structure of the retina [16], it remains unclear whether sellar tumors affect fundus blood flow. Laser speckle flowgraphy (LSFG) is a noninvasive method for evaluating fundus blood flow with good reproducibility, and is useful in various diseases, such as glaucoma [17], ocular ischemic syndrome [18], and ischemic optic neuropathy [19]. In this study, we evaluated the characteristics of fundus blood flow in tumors of the sellar region. We analyzed changes in fundus blood flow before and after tumor resection and investigated the association between fundus blood flow and clinical parameters.

## 2. Materials and Methods

This study followed the guidelines of the Declaration of Helsinki and was approved by the Institutional Review Board of Gunma University Graduate School of Medicine (HS2022-200). An opt-out informed consent protocol was used for this study.

We retrospectively reviewed 54 patients with sellar tumors who underwent endoscopic transsphenoidal tumor resection at Gunma University Hospital between July 2021 and April 2024 and underwent complete ophthalmological examinations both preoperatively and postoperatively. Among these, we analyzed 16 cases (32 eyes) with optic nerve bending in only 1 eye and no optic nerve bending in the other eye. MRI of the sellar and suprasellar lesions in all patients was performed using a 1.5-T or 3-T MRI system. The presence or absence of optic nerve bending was determined using the method reported previously [6], based on the sagittal optic nerve-canal bending angle (ONCBA) measured by MRI before tumor resection surgery. ONCBA refers to the angle created between the optic nerve within the optic canal and the optic nerve in the intracranial subarachnoid space at the entrance of the optic canal. ONCBA was obtained by two neurosurgeons measuring the extent of this bending on sagittal MRI. Each evaluation was conducted by neurosurgeons with over 20 years of experience in pituitary tumor MRI interpretation and surgery. Disagreements were resolved by consensus. Optic nerve bending (large ONCBA) was defined as ONCBA ≥ 45°, and non-optic nerve bending (moderate ONCBA) was defined as ONCBA < 45°, as previously reported [6]. The exclusion criteria were a history of glaucoma or evident glaucomatous optic neuropathy, high myopia (spherical equivalent < −6 diopters), presence of retinal diseases, severe cataract, and poor visualization of the optic nerve on MRI.

All patients underwent ophthalmologic examinations, including measurement of best-corrected visual acuity (BCVA), intraocular pressure refraction, slit-lamp biomicroscopy, color fundus photography (Canon CX-1; Canon Inc., Tokyo, Japan), GCL + IPL thickness using Cirrus high-definition optical coherence tomography (HD-OCT) (Carl Zeiss Meditec, Dublin, CA, USA), visual field testing, and LSFG in both eyes with and without optic nerve bending before and 1 month after tumor resection surgery. BCVA was recorded as the logarithm of the minimum angle of resolution (logMAR) units. The Cirrus HD-OCT ganglion cell analysis algorithm segmentally divided the macula into six regions: the superior, superior nasal, inferior nasal, inferior, inferior temporal, and superior temporal sectors, and subsequently quantifies the combined thickness of GCL + IPL [20]. Visual field testing was conducted using the Goldmann test, and impairment was quantified using the previously reported simple visual field impairment score (SVFIS) [21]. The Goldman perimetry chart was categorized into 12 regions, with lines connecting the upper and lower parts and the nasal and temporal sides within 5°, 5–30°, and >30° of the centers. Points were added if any scotomas were present in each area, including the expansion of physiological scotomas. If the peripheral visual field was narrowed by >10° from the normal range, peripheral visual field impairment was added. Thus, 0 points indicated no visual field impairment, and 12 points indicated visual field impairment in all areas. The evaluation of blood flow in the optic nerve head (ONH) was based on the measurement of the mean blur rate (MBR) using an LSFG-NAVI device (Softcare Co., Ltd., Fukutsu, Japan) with a dilated pupil. Mydrin-P (0.5% tropicamide + phenylephrine hydrochloride) was used to dilate the pupils. LSFG uses a diode laser with a wavelength of 830 nm to illuminate the circulating erythrocytes. The reflected light generated a speckle pattern, which was subsequently utilized to calculate blood flow values, known as the MBR. An elliptical region was manually delineated around the ONH to define the region of interest (ROI) in the composite ocular blood flow map generated by LSFG-NAVI. An experienced operator determined the ROI with elliptical bands of ONH margins in all participants to ensure that the ONH was not over- or under-covered, referring to the fundus photographs. Regarding ONH measurements, the software (LSFG ANALYZER, ver.3.7.0.4; commercially available product) automatically categorized the ROI into large vascular and tissue regions and determined the MBR in three regions: the vascular region ONH (mean of vascular area; MV), tissue region ONH (mean of tissue area; MT), and overall ONH (mean of all area; MA). MV represents the flow in large vessels supplying the retina, MT represents tissue blood flow in the ONH, and MA is a combination of both values.

For statistical analyses, data are presented as the mean ± standard deviation or median (interquartile range). The Mann–Whitney U test or unpaired *t*-test was conducted to compare the BCVA, SVFIS, GCL + IPL thickness measurements, and optic nerve head mean blur rate (ONH-MBR) between the bending and non-bending eyes, as appropriate. The Wilcoxon signed-rank test was conducted to compare changes in BCVA, SVFIS, GCL + IPL thickness, and ONH-MBR before and 1 month after tumor resection. The correlations between BCVA, SVFIS, GCL + IPL thickness, ONH-MBR, and ONCBA were analyzed using Spearman’s correlation coefficient. A significance level of *p* < 0.05 was set. Statistical analyses were performed using GraphPad Prism, version 10 (GraphPad Software Inc., La Jolla, CA, USA).

## 3. Results

Table 1 shows the demographic and clinical characteristics of patients with sellar tumors.

**Figure 1:**
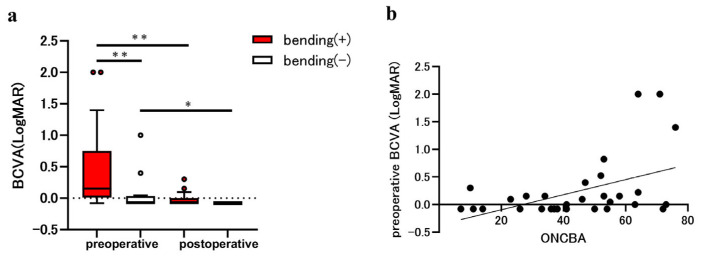
Evaluation of best-corrected visual acuity (BCVA) in eyes with the sellar lesion. The parameters for eyes with optic nerve bending (red bars) and eyes without optic nerve bending (white bars) were compared before and after the tumor resection. (**a**) Comparison of BCVA in eyes with and without optic nerve bending pre- and post-tumor resection. The preoperative BCVA in eyes with optic nerve bending was significantly worse than that in those without optic nerve bending (** *p* < 0.01). The postoperative BCVA was significantly improved in eyes with and without optic nerve bending (** *p* < 0.01 and * *p* < 0.05). (**b**) Graphs showing the correlation between the optic nerve-canal bending angle (ONCBA) and preoperative BCVA in 32 eyes of 16 patients. The ONCBA was positively correlated with preoperative BCVA (r = 0.43, *p* = 0.013).

Nine patients had a PitNET, three had a craniopharyngioma, one had a Rathke’s cleft cyst, and three had a meningioma. Among the patients with sellar and suprasellar tumors, the average age was 58.9 ± 10.7 years, with 11 men (68.8%) and 5 women (31.3%). The mean ONCBA was 60.2 ± 9.7° in the bending eyes and 27.1 ± 11.7° in non-bending eyes (*p* < 0.0001). Preoperative and postoperative BCVAs were 0.15 (0.01 to 0.75) and −0.08 (−0.08 to 0.00) in the bending eyes and −0.08 (−0.08 to 0.03) and −0.08 in the non-bending eyes (Figure 1a). At preoperative examination, the BCVA of the bending eyes was significantly worse than that of the non-bending eyes (*p* = 0.003). The postoperative BCVA was significantly improved compared with the preoperative BCVA in the bending and non-bending eyes (*p* = 0.003 and *p* = 0.03, respectively; Figure 1a). Spearman’s correlation (r = 0.43, *p* = 0.013) showed that ONCBA positively correlated with preoperative BCVA in 32 eyes of 16 patients (Figure 1b).

**Figure 2:**
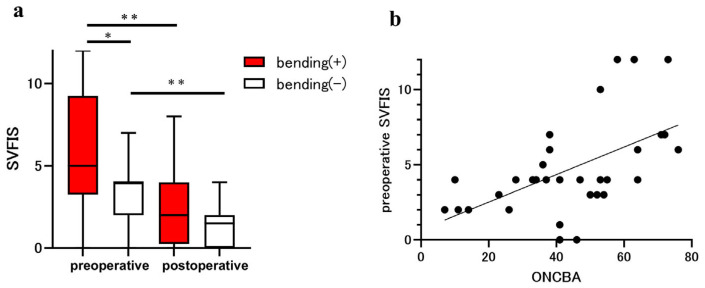
Assessment of visual field using the simple visual field impairment score (SVFIS) in patients with sellar and suprasellar tumors. The parameters for eyes with optic nerve bending (red bars) and eyes without optic nerve bending (white bars) were compared before and after the tumor resection. (**a**) Comparison of SVFIS between eyes with and without optic nerve bending pre- and post-surgery. The preoperative SVFIS of eyes with optic nerve bending was significantly higher than that in those without optic nerve bending (* *p* < 0.05). Postoperative SVFIS was significantly improved in eyes with and without optic nerve bending (** *p* < 0.01). (**b**) Graphs showing the correlation between the optic nerve-canal bending angle (ONCBA) and preoperative SVFIS in 32 eyes of 16 patients. The ONCBA was positively correlated with preoperative SVFIS (r = 0.56, *p* < 0.001).

Preoperative and postoperative SVFIS were 5.00 (3.25 to 9.25) and 2.00 (0.25 to 4.00) in the bending eyes and 4.00 (2.00 to 4.00) and 1.50 (0.00 to 2.00) in the non-bending eyes (Figure 2a). In the preoperative examination, the SVFIS was significantly worse in the bending eyes than in the non-bending eyes (*p* = 0.03). Postoperative SVFIS was significantly improved relative to preoperative SVFIS in the bending (*p* = 0.0011; Figure 2a) and non-bending (*p* = 0.004; Figure 2a) eyes. Spearman’s correlation (r = 0.56, *p* < 0.001) showed that ONCBA was positively correlated with the preoperative SVFIS in 32 eyes of 16 patients (Figure 2b).

**Figure 3:**
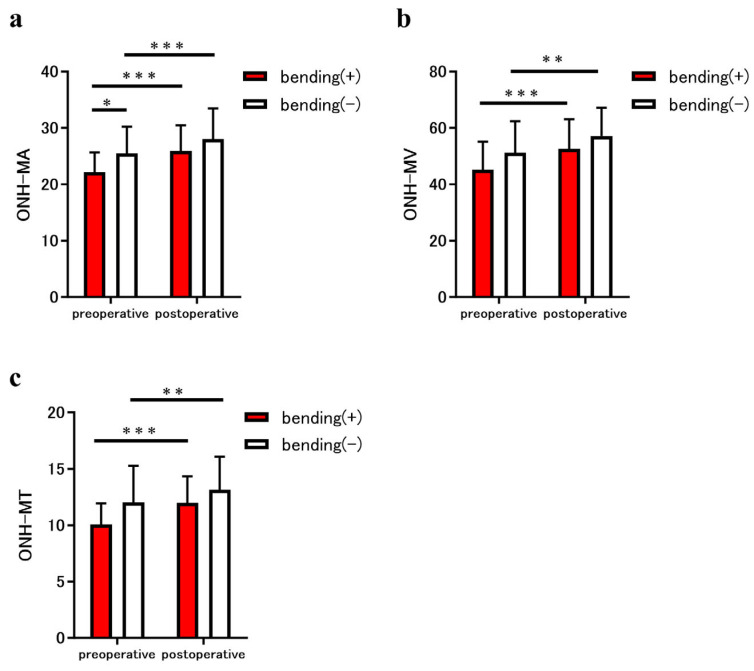
Comparison of the mean blur rate (MBR) measured using laser speckle flowgraphy (LSFG) between eyes in patients with the sellar and suprasellar tumor. The graphs show the optic nerve head (ONH)-MBR of the overall region (MA) (**a**), vessel region (MV) (**b**), and tissue region (MT) (**c**), measured using LSFG. The parameters for eyes with optic nerve bending (red bars) and eyes without optic nerve bending (white bars) were compared before and after the tumor resection. The ONH-MA in eyes with optic nerve bending was significantly lower than that in eyes without bending (* *p* < 0.05). In addition, the preoperative ONH-MA, MV, and MT in eyes with optic nerve bending increased significantly after tumor resection (all *** *p* < 0.001). Similarly, the ONH-MA, MV, and MT of eyes without optic nerve bending increased significantly after tumor resection (*** *p* < 0.001, MA; ** *p* < 0.01, MV and MT).

The MBR parameters of the ONH in both groups are shown in Figure 3. The preoperative MA, MV, and MT of the optic nerve bending group were 22.2 ± 3.5, 45.2 ± 10.0, and 10.1 ± 1.9, respectively, and those of the non-bending group were 25.5 ± 4.8, 51.2 ± 11.2, and 12.0 ± 3.3, respectively. In addition, the postoperative MA, MV, and MT for the optic nerve bending group were 25.9 ± 4.6, 52.6 ± 10.6, and 12.0 ± 2.4, respectively; for the non-optic nerve bending group, they were 28.0 ± 5.5, 57.1 ± 10.0, and 13.2 ± 2.9, respectively (Figure 3a–c). The preoperative MA of the optic nerve bending group was significantly lower than that of the non-optic nerve bending group (*p* = 0.04; Figure 3a). The postoperative MA, MV, and MT in the optic nerve bending group were significantly increased after tumor resection (all *p* < 0.001; Figure 3a–c). Furthermore, the postoperative MA, MV, and MT in the non-optic nerve bending group were significantly increased after tumor resection (*p* < 0.001, MA; *p* = 0.006 and *p* = 0.002, MV and MT; Figure 3a–c).

In preoperative examination, GCL + IPL thicknesses in the bending and non-bending eyes were 67.0 ± 14.2 µm and 72.6 ± 7.9 µm in the superior sector, 66.1 ± 13.6 µm and 69.9 ± 9.8 µm in the superior nasal sector, 64.3 ± 11.6 µm and 68.0 ± 9.2 µm in the inferior nasal sector, 65.9 ± 12.7 µm and 71.2 ± 7.1 µm in the inferior sector, 75.5 ± 10.2 µm and 78.8 ± 7.4 µm in the inferior temporal sector, and 72.3 ± 11.9 µm and 77.7 ± 6.6 µm in the superior temporal sector, respectively. In postoperative examination, GCL + IPL thicknesses in the bending and non-bending eyes were 68.5 ± 9.1 µm and 72.1 ± 7.2 µm in the superior sector, 66.9 ± 10.7 µm and 69.1 ± 9.6 µm in the superior nasal sector, 64.8 ± 10.3 µm and 67.3 ± 8.9 µm in the inferior nasal sector, 67.7 ± 8.4 µm and 71.1 ± 6.7 µm in the inferior sector, 75.4 ± 9.0 µm and 78.2 ± 7.1 µm in the inferior temporal sector, and 73.3 ± 9.4 µm and 77.3 ± 6.3 µm in the superior temporal sector, respectively. No significant differences were observed between the optic nerve bending and non-bending groups in any sector of the GCL + IPL before and after tumor resection. In both bending eyes and non-bending eyes, the thickness of GCL + IPL in the nasal sectors was thinner than that in the temporal sectors both preoperatively and postoperatively. The postoperative SVFIS showed a negative correlation with preoperative GCL + IPL in five sectors aside from the inferior temporal sector (r = −0.65, *p* < 0.001, superior; r = −0.55, *p* = 0.001, superior nasal; r = −0.51, *p* = 0.003, inferior nasal; r = −0.53, *p* = 0.002, inferior; r = −0.17, *p* = 0.34, inferior temporal; and r = −0.38, *p* = 0.03, superior temporal sector).

We further investigated the relationship between the blood flow in the optic nerve head (ONH-MA), measured using LSFG, and other parameters (ONCBA, GCL + IPL, BCVA, and SVFIS). The correlations between the preoperative ONH-MA and other parameters are shown in Table 2.

Preoperative ONH-MA was negatively correlated with ONCBA (r = −0.56, *p* < 0.001). The correlations between postoperative ONH-MA and other parameters are shown in Table 3.

**Figure 4:**
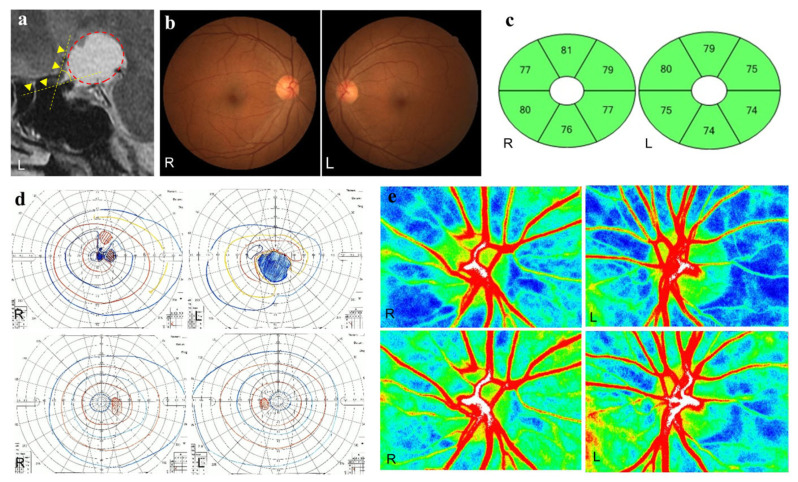
A representative case of craniopharyngioma. A 57-year-old woman has optic nerve bending in her left eye caused by craniopharyngioma. Preoperative and postoperative best-corrected visual acuities (BCVAs; logarithm of the minimum angle of resolution units) are 0.30 and −0.08 in the right eye and 2.0 and −0.08 in the left eye, respectively. (**a**) Sagittal T2-weighted magnetic resonance images before surgery. The optic nerve-canal bending angle (ONCBA) (yellow dotted lines) is formed by the optic nerve in the optic canal and the optic nerve in the intracranial subarachnoid space at the optic canal’s exit. The yellow arrowheads indicated the optic nerve. A circle surrounding red dots indicates the tumor. The ONCBAs of this case are 10° in the right eye and 63° in the left eye. (**b**) Color fundus photograph of both eyes showing the normal appearance at the preoperative visit. (**c**) The thickness of the ganglion cell layer (GCL) + inner plexiform layer (IPL) in both eyes after tumor resection is within the normal range. (**d**) Goldmann perimetry before and after tumor resection. A central scotoma is detected in both eyes before tumor resection. The preoperative SVFIS score for the left eye is lower than that for the right eye (4 points for the right eye; 12 points for the left eye) (**upper** panels). After tumor resection, the central scotoma has disappeared in both eyes, and the SVFIS score is 0 for both eyes (**lower** panels). (**e**) Laser speckle flowgraphy (LSFG) images of the optic nerve head (ONH) before and after tumor resection. The preoperative optic nerve head-mean blur rate (OHN-MBR) of the overall region (MA), vessel region (MV), and tissue region (MT) are 23.1, 46.3 and 11.9 in the right eye, and 22.2, 37.2 and 9.3 in the left eye, respectively (**upper** panels). Postoperative MA, MV and MT for the right eye are 27.9, 54.9 and 12.0; for the left eye, they are 29.6, 55.0 and 11.3, respectively (**bottom** panels).

Postoperative ONH-MA positively correlated with the thickness of the GCL + IPL in the superior (r = 0.39, *p* = 0.03), superior nasal (r = 0.49, *p* = 0.005), inferior nasal (r = 0.42, *p* = 0.02), and inferior regions (r = 0.35, *p* = 0.047). A representative case of a patient with craniopharyngioma and optic nerve bending in the left eye is shown in Figure 4.

## 4. Discussion

We retrospectively analyzed the clinical features of sellar tumors with and without optic nerve bending, focusing on changes in fundus blood flow before and after tumor resection. In eyes with sellar tumors causing optic nerve bending, the ONH blood flow, measured using LSFG, was lower than that in the eyes without optic nerve bending, and significantly improved after tumor resection in both groups. Furthermore, preoperative ONH blood flow was associated with ONCBA, whereas postoperative ONH blood flow was associated with nasal GCL + IPL thickness.

Our group previously reported that sellar tumors can severely impair vision by compressing the optic chiasm and bending the optic nerve, as measured by the sagittal angle at the optic canal entrance on MRI [10]. Although visual impairment due to optic nerve bending is a relatively new concept, another group has recently reported that optic nerve bending is related to visual impairment [12]. This study showed that ONCBA is associated with BCVA and visual field defects. To treat sellar lesions, tumor resection is required before irreversible visual impairment occurs. Optic nerve bending may serve as a key indicator for determining the need for tumor resection. The optic nerve at the entrance of the optic canal is mainly supplied with blood from the superior pituitary artery and does not receive blood from the ophthalmic artery, making it more susceptible to ischemia than the blood-rich optic chiasm [22]. In addition, the optic nerve undergoes demyelination 2 days after compression [23]. Continued compression of the optic nerve can slowly cause irreversible visual impairment despite partial remyelination [23]. Optic nerve bending at the entrance of the optic canal in the narrow peripheral optic nerve may cause more damage to the entire optic nerve than optic chiasm compression in the relatively strong and large optic chiasm [10]. Therefore, optic nerve bending is more likely to cause ischemia and stronger demyelination than optic chiasm compression, and is also more likely to cause severe visual impairment.

LSFG can non-invasively and efficiently evaluate ocular blood flow and has been reported to be useful in differentiating between open-angle glaucoma, optic neuritis, and ischemic optic neuropathy [24]. No prior reports have observed fundus blood flow using LSFG in patients with sellar tumors. This study showed that postoperative ONH blood flow is associated with nasal GCL + IPL thickness. Furthermore, the ONH blood flow improved after tumor resection in the optic nerve bending and non-bending eyes. The nasal GCL + IPL thickness was reduced in patients with PitNET compared to normal participants [25]. This is because compression of the optic chiasm by the tumor damages the crossing fibers, causing retrograde damage to the retinal ganglion cells. A prior OCTA study found reduced retinal vascular density in regions where the retinal ganglion cell layer had thinned, consistent with optic chiasm compression from PitNET [16]. This mechanism involves tumor-induced optic chiasm compression, leading to ganglion cell damage, reduced metabolic activity, and lower retinal nutritional demand [26]. In response, blood flow to the retina decreases, causing changes in the vascular structure of the corresponding area of the retina [27]. One month after tumor resection, the transient functional impairment of retinal ganglion cells caused by optic nerve bending and chiasm compression resolved. Therefore, postoperative ONH blood flow may be related to the original damage to retinal ganglion cells. The improvement in ONH blood flow observed even in eyes without optic nerve bending may be due to the removal of optic chiasm compression by tumor resection. A study that observed the superficial and deep retinal capillary plexus in eyes with PitNET using OCTA reported a significant increase in retinal vascular density after tumor resection [13]. Despite differences between OCTA and LSFG, these findings suggest that sellar lesions cause changes in fundus blood flow and retinal vascular structure before and after surgery. Although postoperative ophthalmic evaluation in this study was performed only 1 month after surgery, GCL + IPL thickness changes due to retrograde degeneration may change for more than 3 months after optic nerve decompression [28,29]. Long-term observation of sellar lesions with optic nerve bending and chiasm compression may reveal further changes in ONH blood flow as well as GCL + IPL thickness. In addition, GCL + IPL thinning in optic nerve-bending eyes was reported to be associated with poor postoperative BCVA [11]. This study showed a correlation between preoperative GCL + IPL thickness in five sectors and postoperative SVFIS. In the treatment of sellar lesions, GCL + IPL thickness may be an important predictor of postoperative visual function.

This study showed that the preoperative ONH blood flow was associated with ONCBA. Optic nerve bending causes reversible retinal ganglion cell axonal dysfunction in the short term; therefore, a preoperative decrease in ONH blood flow may reflect retinal ganglion cell dysfunction. In addition, the optic nerve around the entrance of the optic canal is adjacent to the internal carotid artery (ICA) and ophthalmic arteries. PitNET, regardless of whether or not stroke is present, may cause direct compression of the ICA [30,31]. Previous studies suggest a link between optic nerve bending angle and tumor size [10,11], indicating that severe bending may anatomically affect the arterial system around the optic nerve. However, because the MRI methods used in this study did not identify any changes in the vascular structure caused by the tumor, further investigation of the MRI method is required to clarify this hypothesis.

This study has several limitations, including its retrospective, single-center design and relatively small sample size. To generalize the results of this study, further evaluation of a larger number of cases at multiple institutions is needed. Moreover, this study did not examine the period from the onset of visual impairment to ophthalmological evaluation. The time from the onset of optic nerve bending and optic chiasm compression may affect visual impairment and GCL + IPL thickness, but it was challenging to identify the onset time in this study. This study includes patients with four different types of tumors (PitNET, craniopharyngiomas, meningiomas, and Rathke’s cleft cysts). These tumors differ in their growth patterns, rates, and tissue characteristics, and may have different effects on the optic nerves and surrounding vasculature. However, since they are common in terms of sellar lesions with optic nerve bending and optic chiasm compression, the effect on the results of this study is minimized.

In conclusion, sellar tumors with optic nerve bending and chiasmal compression reduce optic blood flow, which improves after tumor resection. The preoperative optic blood flow was associated with the bending angle of the optic nerve, and postoperative optic blood flow was associated with nasal GCL + IPL thickness. LSFG may be used to evaluate the fundus hemodynamics of PitNET and other sellar regions.

## Figures and Tables

**Table 1:** Systemic characteristics of patients with sellar tumors.

	Participant (*n* = 16)		
	Optic Nerve Bending (+)	Optic Nerve Bending (−)	*p*-Value
Number of eyes (*n*)	16	16	
Age (years)	58.9 ± 10.7		
Male–Female (*n*)	11:5		
Right eye (*n*)	8	8	1
ONCBA (°)	60.2 ± 9.7	27.1 ± 11.7	<0.0001
Pathology			
Pituitary neuroendocrine tumor	9		
Craniopharyngioma	3		
Rathke’s cleft cyst	1		
Meningioma	3		

ONCBA: optic nerve-canal bending angle.

**Table 2:** Association between preoperative optic disc blood mean blur rate (all areas) and other measurements in eyes with sellar tumors.

	R	*p*-Value
ONCBA	−0.56	<0.001
Preoperative examination		
GCL + IPL		
Superior	−0.10	0.59
Superior nasal	−0.07	0.72
Inferior nasal	−0.06	0.75
Inferior	−0.10	0.60
Inferior temporal	−0.18	0.33
Superior temporal	−0.22	0.23
BCVA (LogMAR)	−0.16	0.38
SVFIS	−0.18	0.33

ONCBA—optic nerve-canal bending angle; GCL + IPL—ganglion cell layer + inner plexiform layer; BCVA—best-corrected visual acuity; SVFIS—simple visual field impairment score.

**Table 3:** Association between postoperative optic disc blood mean blur rate (all areas) and other measurements in eyes with sellar tumors.

	R	*p*-Value
ONCBA	−0.27	0.14
Postoperative examination		
GCL + IPL		
Superior	0.39	0.03
Superior nasal	0.49	0.005
Inferior nasal	0.42	0.02
Inferior	0.35	0.047
Inferior temporal	0.13	0.47
Superior temporal	0.16	0.39
BCVA (LogMAR)	−0.01	0.97
SVFIS	−0.10	0.59

ONCBA—optic nerve-canal bending angle; GCL + IPL—ganglion cell layer + inner plexiform layer; BCVA—best-corrected visual acuity; SVFIS—simple visual field impairment score.

## Data Availability

All data relevant to this study are presented in the published manuscript. Additional data supporting the study’s conclusions can be obtained by contacting the corresponding author.

## Abbreviations

The following abbreviations are used in this manuscript:

PitNET	Pituitary Neuroendocrine Tumor.
MRI	Magnetic Resonance Imaging.
GCL + IPL	Ganglion Cell Layer plus Inner Plexiform Layer.
OCTA	Optical Coherence Tomography Angiography.
LSFG	Laser Speckle Flowgraphy.
ONCBA	Optic Nerve-Canal Bending Angle.
BCVA	Best-Corrected Visual Acuity.
OCT	Optical Coherence Tomography.
SVFIS	Simple Visual Field Impairment Score.
ONH	Optic Nerve Head.
MBR	Mean Blur Rate.
ROI	Region of Interest.
MV	Mean of Vascular Area.
MT	Mean of Tissue Area.
MA	Mean of All Areas.
ICA	Internal Carotid Artery.
